# Exploring the Implementation of Early Mobilization of Stroke Patients in Qatar

**DOI:** 10.7759/cureus.92798

**Published:** 2025-09-20

**Authors:** Jidesh Viswambharan, Shahir M Thahira, Sujatha Joseph, Soraya Maart, Linzette D Morris, Gillian D Ferguson

**Affiliations:** 1 Division of Physiotherapy, Department of Health and Rehabilitation Sciences, University of Cape Town, Cape Town, ZAF; 2 Physiotherapy Department, Hamad General Hospital, Hamad Medical Corporation, Doha, QAT; 3 Nursing Department, Hamad General Hospital, Hamad Medical Corporation, Doha, QAT; 4 Department of Rehabilitation Sciences, College of Health Sciences, Qatar University, Doha, QAT

**Keywords:** early mobilisation, feasibility, functional training, physiotherapy, referral to physiotherapy, stroke

## Abstract

Introduction

Providing early rehabilitation services is an integral part of an acute stroke unit. Research studies suggest that early mobilization (EM) of acute stroke patients may be effective in improving functional status and preventing secondary complications. To provide this comprehensive care of EM, several factors need to be fulfilled. The study aims to describe the contextual factors associated with the implementation of EM at a tertiary academic hospital setting in Qatar.

Methods

A retrospective descriptive study design was used. All ischemic male and female patients over the age of 18 years with a first or recurrent stroke who were admitted during the four-month study period at Hamad General Hospital were included. The demographic and clinical profile, time of stroke onset and admission, time of physiotherapist evaluation, and mode of arrival to hospital were collected.

Results

During the study period, 316 ischemic stroke patients were admitted; 79% were male, and the mean (SD) age of the group was 54.2 ± 14.4 years. The most used mode of arrival to the hospital was ambulance service (71%). A total of 33.9% of patients reached the hospital within 4.3 hours of stroke onset. At the facility, the multidisciplinary care, stroke pathway, timely referral to physiotherapy, and follow-up help in effective care.

Discussion

This study presents the clinical profile of ischemic stroke patients admitted to the facility and examines the contextual factors influencing the implementation of EM within this healthcare setting. One key factor affecting EM implementation among ischemic stroke survivors is the timely arrival of patients to the facility. The early referral system for all ischemic stroke patients facilitates the physiotherapy assessment within 48 hours of admission. Additionally, daily coordinated multidisciplinary meetings ensure the effective functioning of stroke care. The findings indicate that this setting provides a suitable environment for EM of acute stroke patients, demonstrating that implementation of EM is feasible and can serve as a model for other units.

## Introduction

Stroke is the second leading cause of death and the third leading cause of disability globally [1]. Early post-stroke complications include fever, aspiration pneumonia, recurrent stroke, myocardial infarction, urinary tract infection, deep venous thrombosis, pressure sores, and falls. Each of these complications can delay the recovery process and extend rehabilitation. The burden of disability due to stroke is significant. Many survivors experience challenges in daily activities, leading to decreased quality of life for themselves and their caregivers [2]. Timely rehabilitation interventions provided by therapists working in multidisciplinary teams are thus essential to improve post-stroke outcomes [3].

Research studies suggest that early mobilization (EM) of acute stroke patients may be effective in improving functional status and preventing secondary complications [3]. Although the term "EM" is variably defined in literature, it commonly means a distinctive characteristic of care delivered within 48 hours of stroke onset. It consists of mobilizing the patient using a functional approach, including activities such as sitting up, getting out of bed, standing, and walking early after stroke and continuing at frequent intervals, between 15 and 45 minutes once, twice, or three times a day to promote recovery [4]. The Canadian Best Practice Guidelines for Acute Stroke Management (2018) suggest that the physiotherapy (PT) evaluation should be conducted within 48 hours of the patient's admission, and these evaluations are recommended to include assessment of patient impairments, mobility, and functional status [5]. Similarly, the National Clinical Guidelines for Stroke for the United Kingdom and Ireland recommend assessing patients with stroke to determine the most appropriate and safe methods of transfer and mobilization. It is further suggested that stroke patients who have difficulty moving and are medically stable should be offered frequent, short daily mobilization within 24 to 48 hours of stroke onset [6]. These sessions should be offered by appropriately trained staff. To provide this comprehensive care, several factors need to be fulfilled. First, the stroke patient should reach the hospital early after having had a stroke. Second, they must complete the admission and diagnostic procedures, receive immediate medical management, and finally, be referred to PT. All this needs to be completed within 48 hours to provide EM [5].

Another important factor to be considered is that the hospital should have a dedicated ward to admit all stroke patients, as this can provide a package of coordinated multidisciplinary care. Dedicated stroke units are an important part of acute stroke care interventions and services, and studies have demonstrated that patients receiving care in these units have better outcomes in terms of mortality and disability compared to patients admitted to general medical wards [7].

Hemorrhagic strokes are associated with a greater average initial stroke severity. Decreased levels of consciousness, surgical intervention, hemodynamic instability, and hematoma growth limit the EM of patients with acute hemorrhagic stroke [8]. Ischemic stroke patients are thus better suited for EM compared to hemorrhagic stroke [9].

A quality improvement project to enhance functional independence following cardiothoracic surgery was conducted in Qatar. The implementation of an early activity and mobilization program resulted in a significant improvement in patients’ first out-of-bed mobilization time, as well as improved mobility and functional independence scores on transfer out of the cardiothoracic intensive care unit. [10]. This study reported that referring patients to PT only post-extubation resulted in mobilization delays due to the absence of a standard referral process. However, implementing referrals to PT at the time of admission, rather than waiting until extubation, led to 100% referral initiation and led to improvement in initiating EM. Since EM and early referral to PT have shown positive effects in this population, their application to stroke patients warrants further exploration.

Integrating evidence-based practices into healthcare services is important to improve patient outcomes. This requires a critical examination of the contextual factors influencing the implementation process. Healthcare providers and managers must consider various patient-related elements, such as hemodynamic stability and reduced level of consciousness. Structural elements refer to staffing limitations, available resources, and availability of mobilization protocols [11]. Process-related barriers include a lack of interdisciplinary collaboration and coordination of services [12]. While EM is a highly recommended practice, the prerequisites for its successful implementation in real-world scenarios are unclear. Thus, the aim of this study was to describe the contextual factors associated with the implementation of EM at a tertiary academic hospital setting in Qatar.

## Materials and methods

Study design

A retrospective descriptive study design was used to collect data from a hospital-based prospective stroke registry.

Study setting

The study was conducted at Hamad General Hospital (HGH), Qatar, a tertiary referral government hospital that admits around 80% of stroke cases in Qatar.

Sampling 

The stroke registry includes all acute stroke admissions to the hospital, from which, convenience sampling, a widely used method in retrospective chart reviews, was undertaken between July and October 2018 to include all patients admitted during this period. The sample reflects a complete list of eligible patients, which reduces selection bias.

Criteria for the study

The medical records of all male and female patients over the age of 18 years with a first or recurrent stroke were included. The records of patients with transient ischemic attack and those who were readmitted for reasons other than a new stroke were excluded.

Ethical approval

Ethical approval for the study was obtained from the Medical Research Centre, Hamad Medical Corporation, Qatar (MRC-01-20-443), and the Human Research Ethics Committee of Faculty of Health Sciences Research, University of Cape Town, South Africa (HREC 451/2020). The data used in this study are anonymized and do not reveal any personal information about patients. The study was conducted according to the Declaration of Helsinki (2013), which promotes and upholds the health, well-being, and rights of patients and regulates the research.

Data collected and analysis

The variables collected from the database were 1) demographic profile of admitted stroke patients, 2) time of stroke onset, admission, and physiotherapist evaluation, and 3) mode of transport to hospital, 4) stroke severity using the National Institutes of Health Stroke Scale (NIHSS), 5) initial presentation, 6) intervention received, and 7) discharge status.

Descriptive statistics are used, including frequencies of categorical data, medians and ranges of ordinal or non-normal numerical data, and means and standard deviations of normally distributed numerical data.

## Results

During the four-month data collection period, 369 stroke patients were admitted, consisting of 316 confirmed ischemic (85%) and 53 hemorrhagic strokes. Among the ischemic stroke patients, 79% were male, and the mean (SD) age of the group was 54.2 ±14.4 years. Female patients were older than male patients, with a mean (SD) age of 62.48 ± 16.58 years compared to 52.01 ± 13 years, respectively. The demographic data, including the nationality of patients, were summarized in Table 1.

**Table 1:** Socio-demographic data of ischemic stroke patients (N=316)

	Frequency (n)	Percentage (%)
Gender		
Male	250	79.1
Female	66	20.9
Nationality		
Bangladesh	45	14.2
Egypt	16	5.1
India	53	16.8
Iran	10	3.2
Nepal	21	6.6
Pakistan	22	7.0
Philippines	34	10.8
Qatar	54	17
Sri Lanka	10	3.2
Sudan	7	2.2
Others	44	13.9
Total	316	100

The most used mode of arrival to the hospital was ambulance service (71%). A total of 33.9% of patients reached the hospital within 4.3 hours of stroke onset, and 13.5% reached it between 4.3 and 12 hours of stroke onset. Twenty patients were admitted with wake-up symptoms, making the exact time of stroke onset difficult to determine. The majority of patients had focal weakness. The stroke onset and arrival time, mode of arrival, and clinical presentation are in Table 2.

**Table 2:** Hospital arrival and clinical characteristics of ischemic stroke patients (N=316)

	Frequency (n)	Percentage (%)
Mode of arrival at the hospital		
Ambulance service	225	71.2
In-hospital stroke	5	1.6
Non-medical transport	78	24.7
Transfer from other hospitals	8	2.5
Time between symptom onset and hospital arrival		
< 4.30 hours	107	33.86
5 – 12 hours	43	13.6%
12-24 hours	20	6.3 %
24-48 hours	47	14.87
48-72 hours	28	8.86 %
>72 hours	37	11.7 %
Wake-up stroke	20	6.33
Onset-unsure	14	4.4
Clinical presentation on admission		
Focal Weakness	228	72.2
Aphasia	18	5.7
Neglect	2	0.6
Ataxia	11	3.5
Diplopia	22	7.0
Dysphagia	3	0.9
Dysarthria	92	29.1
Loss of consciousness	15	4.7
Facial weakness	36	11.4
Dizziness	86	27.2
Headache	43	13.6

On admission, 82% were mild, 9.2% had moderate, and 8.8% had severe strokes in terms of NIHSS severity. Severe stroke patients stayed longer in the hospital (Table 3). 

**Table 3:** Stroke severity and length of stay NIHSS: National Institutes of Health Stroke Scale

NIHSS Classification (0-42)	Male	Female	Total	Percentage (%)	Mean length of stay (days)	SD
Mild (<8)	209	50	259	82	3.8	3.3
Moderate (9-15)	25	4	29	9.2	8.3	7.1
Severe (>16)	16	12	28	8.8	14.9	10.6
Total			316	100%		

The proportion of patients who received interventions like thrombolysis, thrombectomy, carotid revascularization, stenting, hemicraniectomy, and PT referral is in Table 4.

**Table 4:** Interventions performed and PT referral NIHSS: National Institutes of Health Stroke Scale

	Frequency (n)	Percentage (%)
Interventions performed		
Thrombolysis	45	14.2
Thrombectomy	13	4.1
Carotid revascularization	1	0.3
Carotid endarterectomy/Stenting	3	0.9
Hemicraniectomy	3	0.9
Physiotherapy referral		
Patients not referred to PT (NIHSS 0-1)	95	30
Patients referred to PT	221	70

Stroke service

The HGH is a 650-bed facility with a dedicated stroke ward and is accredited by the Joint Commission International for stroke service. The stroke care at HGH is offered through the emergency medical services (EMS), radiology, stroke ward, and intensive care unit. The multidisciplinary team (MDT) at HGH consists of stroke physicians, stroke-trained nurses, and allied health staff, including physiotherapists. The daily MDT meeting, stroke pathway, and policy help in shaping patient treatment plans according to each patient’s individual needs. All eligible patients were referred to and seen by a physiotherapist within 48 hours of admission. Patients with symptoms not relevant to PT, like slurred speech and very mild stroke symptoms, such as numbness, within the NIHSS range of 0-1 (N=95), were not referred to PT. Physiotherapists evaluated those referred up to midday on the same day, and those referred after midday were attended on the next day.

## Discussion

This study presents the profile of ischemic stroke patients admitted to a tertiary hospital in Qatar and discusses some of the contextual factors associated with the implementation of early mobilization in this setting. Approximately 85% of all stroke cases during the study period are ischemic, which has important implications for EM practices. Ischemic strokes, compared to hemorrhagic strokes, are generally more amenable to EM [9]. This suggests that a larger proportion of stroke patients admitted to this tertiary hospital in Qatar may be eligible for EM, potentially enhancing recovery outcomes.

A study published in 2024 examined the annual incidence of stroke in Qatar using the data of stroke patients admitted to Hamad General Hospital from January 2014 to July 2022. The authors reported an incidence of 41 per 100,000 population per annum [13]. The mean age of patients in our study, 54 years, is comparable to the median age of 52 years from another study conducted in Qatar [14]. In contrast, the mean age of patients from a recent European study was reported to be 73.58 (SD 14.25) [15].

Women make up a small proportion of stroke cases, and the higher incidence of stroke among younger people is likely due to the high young male expatriate population that makes up much of the active workforce in Qatar [13]. The burden of non-communicable diseases, such as stroke, is increasing in Qatar, with most patients being male, under the age of 65, and of South Asian, Middle Eastern, or North African descent, as per the studies from these regions [16]. These characteristics are most likely due to the large number of migrant workers that make up the Qatari population.

Most subjects in the study (72%) presented with muscle weakness, which is common in stroke patients. Assessing and addressing these factors is important for safe mobilization, and EM counters the negative effect of bed rest and reduces weakness. A small percentage presented with ataxia and neglect. Aphasia, with a sensory component presenting as a challenge to EM, requires experienced therapists to assist with mobilization. It is interesting that only 5% had loss of consciousness on admission, and EM in this small group is a barrier due to underlying pathology. The data suggest the majority of strokes were mild and can be mobilized early.

This low incidence of severe stroke may be due to more patients and people in Qatar being in a younger age group. A significant percentage present with dizziness, which can impede EM, making it difficult and requiring extra safety precautions to prevent falls.

Structural factors associated with EM

In this study, 71% of ischemic stroke patients reached the hospital using the ambulance service, and 68.67% of them reached the hospital within 48 hours of symptom onset, which enables the physiotherapist to start EM. A significant proportion (53.79%) reached the hospital within 24 hours, a crucial time that makes very early mobilization. In higher-income countries, such as the US, one study reported similar results: just under a third (31.7%) of all stroke patients arrived at the emergency department (ED) within three hours [17], compared to our findings of 33.9% of patients arriving in less than 4.3 hours. Prehospital care provided by EMS plays an important role in improving patient outcomes [18]. From a rehabilitation perspective, the stroke patient should reach the hospital as soon as possible following the onset of the stroke to reduce further brain damage, complete the initial medical management, and start the EM. The findings of this study support the efficiency and accessibility of Qatar’s prehospital emergency health services.

Early arrival time may be a significant barrier in other regions, depending on the geographical location and the availability of stroke services. In addition, studies have identified poor understanding of stroke symptoms and misdirected initial actions as factors that are strongly associated with delayed presentation [19]. There is a disparity in stroke service between high- and low-income countries as per a survey from the Middle East and adjacent countries [20]. For instance, a recent study from South India showed a median prehospital delay of 11.30 hours from stroke onset for 73.3% of patients and a delay of more than 6 hours for 74% of patients [21].

Stroke symptom knowledge and implementation of EM

Knowledge of early warning signs of stroke and the need to access immediate care may be another factor related to the early arrival time [22]. Similarly, a Polish study indicated that the previous occurrence of stroke in a relative or friend impacted knowledge of stroke [22]. In Qatar, the awareness of stroke among the population is appreciable; the effective use of ambulance services, combined with the Arabic cultural emphasis on family-centeredness, which involves a strong commitment to family and mutual support, may contribute to reduced time in arriving at the hospital [23].

Early mobilization and timely referral

To initiate EM, the patients should be referred to PT upon admission. According to the records reviewed, the immediate referral to PT was efficient, and this system helped the therapist to complete the initial evaluation within 48 hours of admission. The availability of dedicated stroke physiotherapists in HGH, the priority given to acute stroke patients, and the effective weekend service also made it easier to evaluate the patients within 48 hours of admission. These factors are written into hospital policy, and adherence is carefully monitored. The PT staff, upon the initial evaluation, plan the intervention accordingly, namely, in-bed exercise or mobilization [24].

In comparison, a study from Nigeria shows that 81% of stroke patients are referred for inpatient PT during admission, and only 63% received PT [25]. Another study from Nigeria shows a mean time from admission to referral for PT was three days [26]. In contrast, in Australia, stroke patients should be assessed by a physiotherapist within 24-48 hours of admission, and this is a part of the acute stroke clinical care standard indicators. The national benchmark in Australia is 92% adherence, and they were able to reach 73% adherence in 2019, 81% in 2021, and 79% in 2023, as per the stroke audit report [27]. A survey from 16 Middle East and North Africa countries shows PT is initiated for stroke within 48 hours of admission in 14 countries [28].

The referral procedures followed at a hospital vary across settings and are often determined by hospital policies. In the current study, all patients who were eligible for PT (i.e., NHSS score >1) were referred to and seen by a physiotherapist within 48 hours of admission. A recent study specifically done to calculate the duration from stroke onset to first PT session from a low-resource setting (Nigeria) with 147 stroke patients showed a delay of 13.7 ± 15.0 days [29]. A scoping review on acute stroke care and rehabilitation services from low- and middle-income countries also shows a delay of three days from admission to referral to rehabilitation services [30]. The referral system in the HGH adheres to guidelines recommending that stroke patients should commence initial evaluation by PT within 48 hours of a stroke; hence, EM is possible here. As 47% of ischemic stroke patients reached the hospital within 12 hours of symptom onset, a proportion of patients were seen by PT within 24 hours of symptom onset. The daily coordinated multidisciplinary meeting at HGH oversees the effective functioning of the stroke care and serves as a model to other units. EM has many benefits, and its implementation requires careful planning.

Study limitations

The study findings are limited to the data captured in the hospital's electronic records by nurses, physiotherapists, and physicians.

## Conclusions

This study looks at the clinical profile of patients admitted with ischemic stroke and contextual factors influencing the implementation of EM within this healthcare setting. Most of the patients were male, making up 79%, and 53.79% of patients reached the hospital within the first 24 hours. All the eligible patients were referred early to PT and evaluated by PT within 48 hours of stroke onset. Starting from the early arrival of stroke patients, referral to PT upon admission, availability of physiotherapists, and priority given for acute stroke patients are the essential factors that enable early assessment and initiation of EM. The care pathway for stroke at this tertiary hospital was determined through hospital policy, and the implementation appears to work well. The findings indicate that this setting provides a suitable environment for EM of acute ischemic stroke patients, demonstrating that implementation of EM is feasible and can serve as a model for other units. Further research related to the impact of EM on acute stroke patients from Middle Eastern hospitals is needed.
